# Nanoantenna-assisted plasmonic enhancement of IR absorption of vibrational modes of organic molecules

**DOI:** 10.3762/bjnano.8.99

**Published:** 2017-05-03

**Authors:** Alexander G Milekhin, Olga Cherkasova, Sergei A Kuznetsov, Ilya A Milekhin, Ekatherina E Rodyakina, Alexander V Latyshev, Sreetama Banerjee, Georgeta Salvan, Dietrich R T Zahn

**Affiliations:** 1 A.V. Rzhanov Institute of Semiconductor Physics, Novosibirsk, 630090, Russia; 2 Novosibirsk State University, Novosibirsk, 630090, Russia; 3 Institute of Laser Physics of SB RAS, Novosibirsk, 630090, Russia; 4 A.V. Rzhanov Institute of Semiconductor Physics RAS, Novosibirsk Branch “TDIAM”, Lavrentiev Ave. 2/1, Novosibirsk, 630090, Russia,; 5 Semiconductor Physics, Technische Universität Chemnitz, D-09107 Chemnitz, Germany

**Keywords:** cobalt phthalocyanine, cortisol, localized surface plasmon resonance, metal nanoantennas, Raman scattering, surface-enhanced infrared absorption (SEIRA)

## Abstract

Nanoantenna-assisted plasmonic enhancement of IR absorption and Raman scattering was employed for studying the vibrational modes in organic molecules. Ultrathin cobalt phthalocyanine films (3 nm) were deposited on Au nanoantenna arrays with specified structural parameters. The deposited organic films reveal the enhancement of both Raman scattering and IR absorption vibrational modes. To extend the possibility of implementing surface-enhanced infrared absorption (SEIRA) for biological applications, the detection and analysis of the steroid hormone cortisol was demonstrated.

## Introduction

Organic semiconductors have been extensively investigated during the past few decades due to their wide range of applications in various organic–inorganic hybrid devices [1]. The ability to tailor the chemical structure of the organic molecules according to the device requirements, in addition to the light weight, flexibility and easy processing of these materials, open up the possibility of fabricating novel hybrid devices [2]. In the last decade, organic semiconductors gained the attention of the spintronics community as these organic semiconductors have been considered as good candidates for spin transport. The most interesting property of organic semiconductors for spintronic applications is the weak spin–scattering mechanism [3], which means that the spin polarization of the carriers can continue for an extended time (in the range from microseconds to milliseconds) [4]. This feature is caused by very low spin–orbit coupling and weak hyperfine interaction.

Phthalocyanines (Pcs) are a class of stable, planar small molecules, often investigated as promising candidates for molecular spintronics [5–6]. There have been studies on molecule/metal interfaces [7], magnetic coupling in the metal phthalocyanine layers [8], spin transport or magnetic properties through single molecules or even thin layers [6,9–10] of phthalocyanines. These molecules also offer the possibility of changing the spin-dependent transport mechanism by slightly modifying the molecular structure [6]. It has been previously shown that the molecular structure of a magnetic material can be probed by various spectroscopic techniques [11]. Under conditions of plasmonic enhancement, a magnetic material can be employed for a wide range of applications [11].

A relatively low optical signal from the vibrational modes of organic molecules using conventional spectroscopic techniques such as infrared (IR) and Raman spectroscopy restricts their detection limit, which is crucial for sensor applications. The sensitivity of these optical methods can be drastically increased by implementation of nanoantenna-assisted plasmonic-enhanced spectroscopy techniques such as surface-enhanced IR absorption (SEIRA) [12] or surfaced-enhanced Raman scattering (SERS) [13]. The principle of SEIRA and SERS is based on specially designed, resonant, metal nanoantennas, providing a high electromagnetic field intensity in close proximity to the plasmonic nanostructure when resonantly excited in the IR or optical regime [14–15]. It was shown that elongated nanoantennas can enhance the SEIRA signal by molecular vibrations in model adsorbates such as octadecanthiol (ODT) [16] and 4,4'-bis(*N*-carbazolyl)-1,1'-biphenyl (CBP) by up to five orders of magnitude [17]. The IR absorption bands of these molecules become pronounced, even for molecular monolayers, by tuning the localized surface plasmon energy of the nanoantennas to the energy of the molecular vibrations. Along with SEIRA, SERS is also traditionally used to study the vibrational spectra of various organic and biological substances [18], which may be present in very low quantities down to single molecules [19]. Raman enhancement up to 10^14^ can be achieved.

Although SEIRA is a relatively new tool for detection of organic and biological substances, it is found to be very effective for probing extremely low concentrations. Adato et al. demonstrated detection of 3 × 10^−19^ moles of silk protein for the entire nanoantenna array, corresponding to only 145 molecules per antenna [20]. A similar approach is used for the terahertz (or far-IR) spectral range for which special nanoscale slot-antenna arrays were designed to determine glucose and fructose in solutions, including market beverages [21]. Terahertz transmittance measurements were made in the frequency range of 0.5–2.5 THz for concentrations from 10 to 4168 mg/dL. Similar structures have been used for the determination of pesticide concentration in solution and on the surface of fruit. The detection limit was 8 μmol [22].

Here, we demonstrate the application of SEIRA for the detection and analysis of vibrational modes of cobalt phthalocyanine deposited on Au nanoantenna arrays. The estimation of the plasmonic enhancement of the fabricated nanoantenna arrays was performed by analyzing the SEIRA and SERS spectra of homogeneous, ultrathin Co Pc films. We also demonstrate SEIRA by detection of the steroid hormone cortisol deposited on Au nanoantenna arrays to extend the possibility of using the method also for biological applications.

## Results and Discussion

### Applications of SEIRA for organic compounds

**Figure 1:**
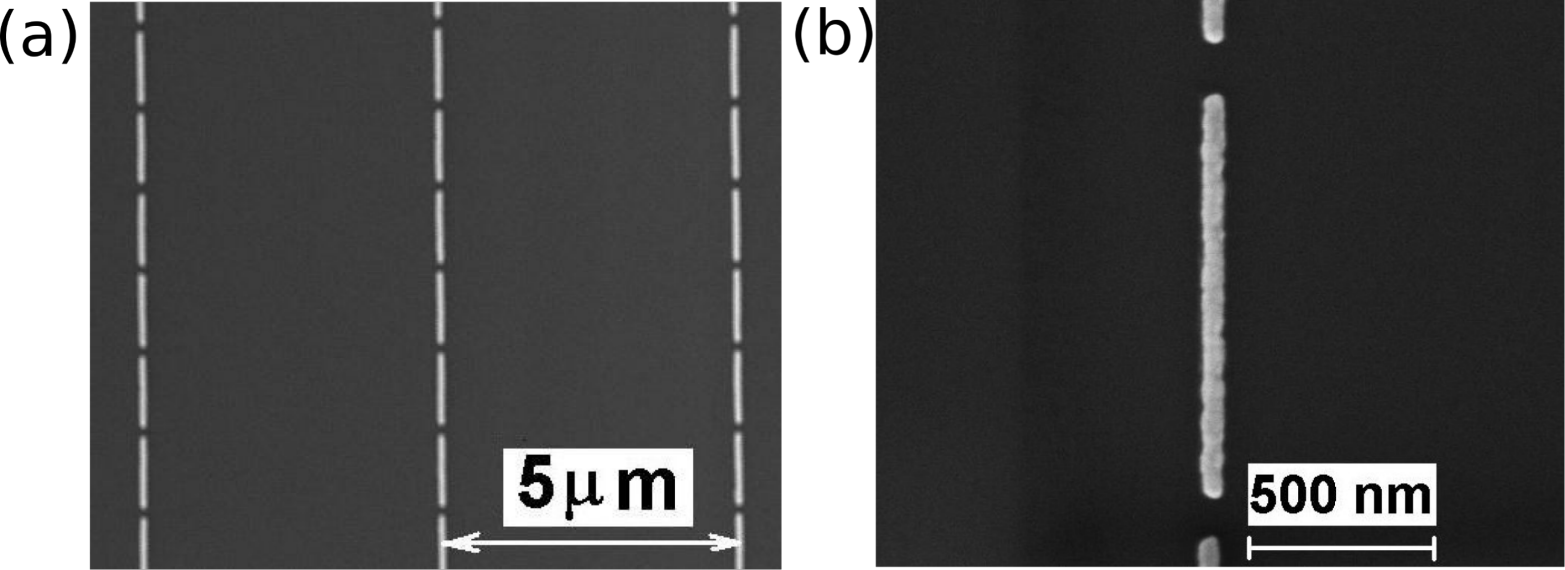
(a) Representative SEM image of Au nanoantenna array; nanoantenna length 900 nm. (b) Detailed image of a nanoantenna.

Representative SEM images of the Au nanoantenna arrays (length 900 nm; width 60 nm) employed for the IR investigation are shown in Figure 1. The period of the array is 5 µm and the distance between nanoantenna edges is about 100 nm.

**Figure 2:**
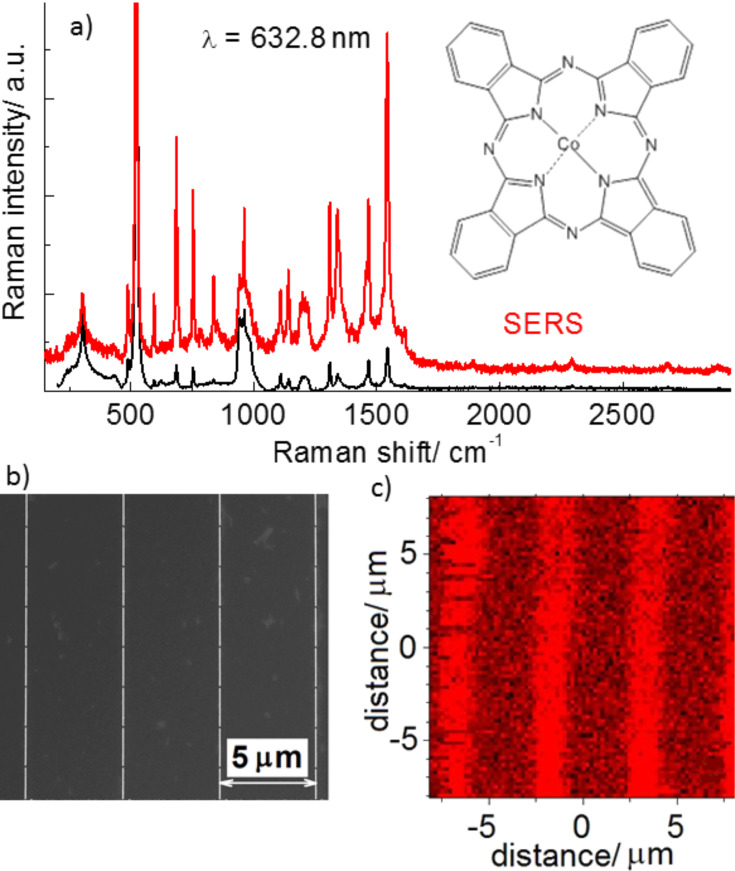
(a) Raman and SERS spectra of a cobalt phthalocyanine (CoPc) film with thickness of 3 nm deposited on a nanoantenna array. The chemical structure of CoPc is shown in the inset. (b) SEM image of the nanoantenna array with antenna length of 1900 nm. (c) Raman mapping (100 × 100 pixels) of the C=N mode (1543 cm^−1^) intensity.

For the quantitative estimation of the nanoantenna-assisted plasmonic enhancement of the fabricated arrays, ultrathin CoPc films (with a thickness up to 3 nm) were deposited on the antenna arrays and a Si substrate. The homogeneity of the CoPc films deposited on the arrays was probed using micro-Raman mapping. The Raman spectra of the CoPc films deposited on a Si substrate (Figure 2a) reveal a rich spectrum of CoPc vibrational modes similar to that observed previously [23]. Note that the excitation energy was 1.96 eV (632.8 nm), which matches well with the HOMO–LUMO gap energy of CoPc (1.9 eV). The coincidence of the excitation energy with that of the electronic transitions in CoPc defines the conditions for resonant Raman scattering (RRS) in CoPc. In addition to the most intense mode of the Si substrate (observed at 520.5 cm^−1^), the RRS spectrum (Figure 2) is dominated by the vibrational mode at 1543 cm^−1^ assigned to the C=N stretching mode [23]. The chemical structure of CoPc is shown in the inset of Figure 2a. The mode frequencies observed at 683, 750, 958, 1307, 1340, and 1465 cm^−1^ are in accordance with the literature data [23].

The Raman spectra measured from CoPc deposited on the Au nanoantennas demonstrate the enhancement of the Raman scattering (a factor of about 9) without noticeable shift of the mode frequencies, manifesting a SERS effect by CoPc. In the case of nanoantennas, the SERS enhancement is much weaker than that determined for CoPc on Au nanocluster arrays (enhancement factor of 2 × 10^4^) observed in our previous experiments [24]. Much stronger SERS enhancement of CoPc on Au nanocluster arrays with respect to that for nanoantennas can be explained by the resonant SERS effect, as the energy of the local surface plasmon resonance (LSPR) of nanoclusters is located in the red spectral region, which is in resonance with the excitation energy. In the case of nanoantennas, the energy of longitudinal LSPR modes polarized along the antennas shifts in the IR spectral range, which leads to off-resonant SERS conditions. The transverse LSPR modes polarized perpendicular to the nanoantennas undergo a small blue shift along with a decrease in the mode intensity [25], which leads to reduced SERS enhancement. Despite this, the SERS effect in CoPc on nanoantenna arrays allows the homogeneity of the CoPc coverage on a nanoantenna array to be investigated using Raman mapping. The intensity of the C=N stretching mode at 1543 cm^−1^ was monitored. The Raman map obtained for a Au nanoantenna array with a 3 nm thick CoPc film shown in Figure 2b,c agrees well with the SEM image of the same structure. One can see from Figure 2c that the Raman mapping indicates the position of the nanoantennas by the stronger Raman (SERS) intensity (brighter regions), which reproduces the 5 µm periodicity of the nanoantenna array and evidences the homogeneous coverage of CoPc over the sample.

**Figure 3:**
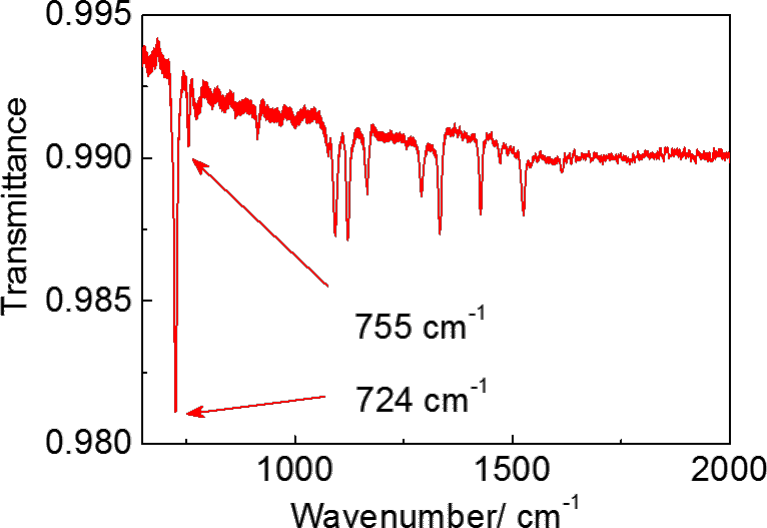
IR transmission spectrum of a 10 nm thick CoPc film deposited on a Si substrate normalized to the IR spectrum of a Si substrate.

The IR spectrum of a 10 nm thick CoPc film is presented in Figure 3 in the spectral range of 500–2000 cm^−1^, where most absorption lines of CoPc are located. The vibrational spectrum of the CoPc film is similar to that defined for CoPc earlier [26–31] by means of IR absorption. The absorption line at 726 cm^−1^ attributed to nonplanar deformation of C–H bonds of benzene rings [26–28] has the strongest intensity and, therefore, was chosen for further SEIRA experiments. It is accompanied by a weaker mode at 755 cm^−1^, referred to as the nonplanar (out-of-plane) bending of C–H bonds and the Co–N bond vibrations [26–28].

**Figure 4:**
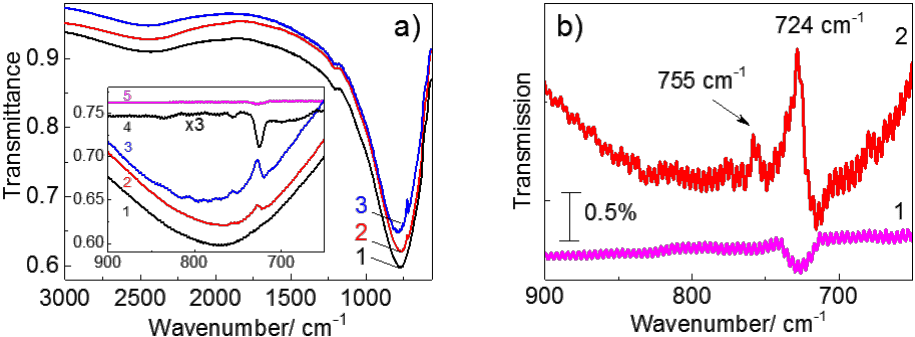
(a) IR spectrum of bare nanoantennas (curve 1) and IR spectra of nanoantennas with deposited 3 nm and 10 nm thick CoPc films (curves 2 and 3, respectively). The inset shows a comparison of the same spectra with the IR spectra of 3 nm and 10 nm thick CoPc films on a Si substrate without nanoantennas (curves 4 and 5, respectively). (b) Comparison of IR spectrum of bare nanoantennas (curve 1) and with the spectrum of the structure with the 3 nm thick CoPc film (curve 2) after background subtraction.

Au nanoantenna arrays with structural parameters (nanoantenna length and period) designed to ensure the LSPR band energy from 600 to 1000 cm^−1^ were fabricated (Figure 4a). In order to determine the structural parameters of the arrays with the targeted LSPR energy and a maximal SEIRA enhancement, 3D full-wave simulations were carried out and the distribution of the electromagnetic field near Au nanoantennas on a silicon substrate was simulated as described in [32]. In the simulations, the same nominal values of nanoantenna width, height, and the spacing between nanoantennas were assumed as imposed by the nanofabrication technology.

Except for the fundamental LSPR mode, the third-order resonance centered at about 2400 cm^−1^ occurs in the IR spectra. A weaker feature near 1200 cm^−1^ is assigned to the surface optical mode from natural silicon oxide covering the Si substrate [33]. The deposition of thin CoPc films on the nanoantenna arrays leads to intensity enhancement for the vibrational modes at 724 and 755 cm^−1^, which are inherent to CoPc in the spectral range of the LSPR band. Note that for the 3 nm thick CoPc film, the mode at 755 cm^−1^ is observed only in the case of the CoPc film deposited on the nanoantenna array (Figure 4a,b). Weak oscillations seen in Figure 4a,b are the interference fringes at the sample thickness (about 400 µm). The overall enhancement factor (EF) for the 10 nm thick CoPc film amounts to 2 and is within the range of 4–5 for a 3 nm thick film. The increase of the EF with decreasing CoPc film thickness is due to the strong electric filed localization near the Si surface in the nanogap between the nanoantenna edges. Using the approach similar to that described in [34], one can quantitatively estimate the enhancement induced by nanoantennas (EF_N_). It can be calculated as the ratio of the areas covered by the nanogaps to the entire nanoantenna array and amounts to about 1/1200. Here, we suppose that the IR absorption predominantly takes place in the nanogap. Taking the maximum EF = 5 for the 3 nm thick CoPc film, the enhancement induced by nanogaps in the array reaches the value of EF_N_ = 6000.

In accordance with previous studies [18,20,35], the vibrational modes observed in the SEIRA spectra exhibit a clear Fano line shape.

### Further applications of SEIRA for biological compounds

A similar approach was used for the investigation of cortisol using SEIRA. Among other organic compounds, the steroid hormones are of great importance because they influence many physiological processes in humans and animals.

**Figure 5:**
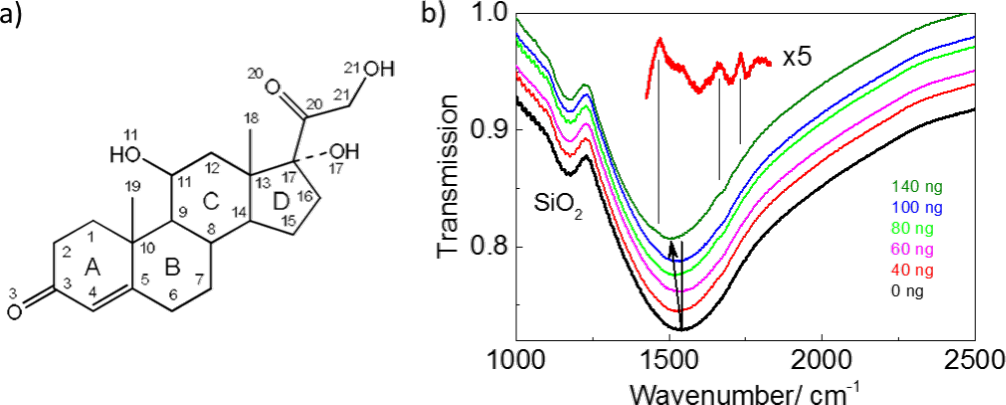
(a) The cortisol chemical structure and numeration of atoms in the cortisol molecule. (b) IR spectrum of a bare Au nanoantenna array and cortisol deposited from the solution with concentration 10ng/µL on the array. Inset shows the detailed fragmentary IR spectrum of 40 ng of cortisol after subtraction of the envelope line.

Cortisol is the major glucocorticoid hormone produced in the adrenal gland and in several tissues and regulates blood glucose levels. Cortisol production disorders lead to the development of diabetes, Cushing's syndrome and other pathologies [36]. Therefore, the determination of the cortisol concentration in blood and tissue is important. The immunoassay methods are predominantly performed in clinical practice [37]. Other methods, such as chromatography [38–40] and surface plasmon resonance [41], are used in fundamental research. Each of these methods of cortisol detection has advantages and drawbacks. Conventional Raman scattering, which is widely used for the investigation of organic and inorganic materials, cannot be applied for investigation of cortisol in blood and tissues due to its low Raman signal response. Therefore, SEIRA is considered as a complementary method for enhanced cortisol detection. Cortisol is the derivative of 1,2-cyclopentanephenanthrene. The molecule core of cortisol consists of four fused carbon rings: three cyclohexane rings (denoted as A, B, and C in the Figure 5a) and one cyclopentane ring (the D ring).

The IR spectrum of cortisol reveals the most pronounced absorption bands associated with valence vibrations of C=O groups [42–43]. According to [44] the calculated (experimental) frequency positions of the bands are located at 1686 (1660) cm^−1^ and 1725 (1735) cm^−1^ and demonstrate the highest intensities. Therefore, the structural parameters of the nanoantennas were chosen in such a way to ensure the LSPR absorption band close to the energy of the most pronounced vibrational modes in cortisol. The deposition of cortisol on a solid substrate implies, as a general rule, the use of the drop-casting method, which results in inhomogeneous coverage. The dielectric function of the media surrounding the nanoantennas is changed after deposition of cortisol solution onto the surface. This results in an LSPR energy shift from 1535 cm^−1^ towards lower energy (near 1500 cm^−1^). Three characteristic vibrational modes of cortisol are also seen in the IR spectrum after subtraction of the envelope line (Figure 5b). These modes can be assigned as deformation vibrations of C–H bonds (1470 cm^−1^), stretching vibrations С3=O3 (1660 cm^−1^) and С20=O20 bonds (1735 cm^−1^). The detection limit of the cortisol concentration determined from the IR spectra was 40 ng. With increasing cortisol concentration (up to 100 ng) the intensity of the modes increases and remains unchanged with further concentration increase. The most probable explanation is that the thickness of the cortisol film reaches the value of the nanoantenna height, which prevents further increase of the optical response. The determined detection limit of the cortisol concentration from the analysis of the SEIRA spectra corresponds to the concentration of the steroid in real biological assays.

## Conclusion

In this work, we characterized the nanoantenna-assisted plasmonic enhancement of IR absorption and Raman scattering from vibrational modes of organic molecules. Au nanoantenna arrays with specified structural parameters were employed to enhance the absorption signatures from the vibrational modes of cobalt phthalocyanine ultrathin films and cortisol molecules. This work may have a wide range of applications as it opens up the possibility to spectroscopically study a magnetic material under the influence of localized plasmonic enhancements, thus increasing the sensitivity limit. As a proposed further application of SEIRA, it was also shown that the detection limit of cortisol using SEIRA corresponds to 40 ng – this opens up the possibility for determination of steroid concentrations in real biological assays.

## Experimental

The uniform periodic arrays of linear Au nanoantennas with length 900 and 1900 nm and period of 5 µm were fabricated on (001)-oriented Si substrates by a direct writing nanolithographic machine (Raith-150, Raith GmbH, Germany) and covers an area of 3 × 3 mm^2^, providing the LSPR band energies in the range from 1580–800 cm^−1^. To avoid interference in the SEIRA spectra, 400 µm thick Si substrates were used. The fabrication process of nanoantenna arrays is very similar to that described in [24] for Au nanocluster arrays.

The structural parameters of nanoantenna arrays were controlled by a scanning electron microscopy (SEM) using the same Raith-150 system at 10 kV acceleration voltage, 30 µm aperture, and 6 mm working distance.

Ultrathin CoPc films with thickness 3 and 10 nm were formed using organic molecular beam vapor deposition onto arrays of Au nanoantennas. The thermal evaporation of the organic molecule was performed in a vacuum chamber at a pressure of ≈5 × 10^−8^ mbar. The evaporation temperature was approximately 400 °C and the deposition rate was ≈0.5 nm/min. During the organic film growth, the substrate was kept at room temperature.

Cortisol (11β)-11,17,21-trihydroxypregn-4-ene-3,20-dione) was purchased from Calbiochem (USA) and was used without further purification. Cortisol dissolved in ethanol at concentration of 10 ng/mL (27.6 μM) was deposited onto arrays of Au nanoantennas by drop-casting. The drop volume was 2 μL.

The LSPR energy on Au nanoantenna arrays with and without organic material was determined from the IR transmission measurements carried out by using a Bruker Vertex 80v Fourier transform infrared spectrometer in the spectral range of 600–4000 cm^−1^. The spectral resolution was 2 cm^−1^ over the whole spectra range. The ratio of the transmission spectra polariszed along and perpendicular to the long axis of the bare nanoantennas and with deposited organic films was analyzed. The noise level was below 0.1% in the IR experiments. The measurements were carried out at room temperature.

Non-polarized Raman spectra were measured using Labram spectrometers equipped with a Renishaw InVia Raman microscope (the laser beam was focused to a spot with a diameter of about 1 µm) in a backscattering geometry at room temperature. A HeNe laser was used as an excitation source at the wavelength of 632.8 nm (2.41 eV). A laser power of less than 100 µW (before the microscope) was used to avoid possible effects of local heating. The spectral resolution was below 2.5 сm^−1^ over the whole spectral range.
